# Molecular targeted therapies for cutaneous squamous cell carcinoma: recent developments and clinical implications

**DOI:** 10.17179/excli2023-6489

**Published:** 2024-02-27

**Authors:** Harpreet Singh, Hitesh Chopra, Inderbir Singh, Sourav Mohanto, Mohammed Gulzar Ahmed, Shruti Ghumra, Anmol Seelan, Manisha Survase, Arvind Kumar, Amrita Mishra, Arun Kumar Mishra, Mohammad Amjad Kamal

**Affiliations:** 1 School of Pharmaceutical Sciences, IFTM University, Moradabad, U.P., India, 244102; 2 Department of Biosciences, Saveetha School of Engineering, Saveetha Institute of Medical and Technical Sciences, Chennai - 602105, Tamil Nadu, India; 3 Chitkara College of Pharmacy, Chitkara University, Punjab, India; 4 Department of Pharmaceutics, Yenepoya Pharmacy College & Research Center, Yenepoya (Deemed to be University), Mangalore, Karnataka, 575018, India; 5 Department of Biological Sciences, Sunandan Divatia School of Science, NarseeMonjee Institute of Management Studies (NMIMS), Pherozeshah Mehta Rd, Mumbai, India, 400056; 6 Mahatma Gandhi Mission, Institute of Biosciences and Technology, Aurangabad, India; 7 School of Pharmaceutical Sciences, Delhi Pharmaceutical Sciences and Research University, New Delhi, India, 110017; 8 SOS School of Pharmacy, IFTM University, Moradabad, U.P., India, 244102; 9 Institutes for Systems Genetics, Frontiers Science Center for Disease-Related Molecular Network, West China Hospital, Sichuan University, China; 10 King Fahd Medical Research Center, King Abdulaziz University, Saudi Arabia; 11 Department of Pharmacy, Faculty of Allied Health Sciences, Daffodil International University, Bangladesh; 12 Enzymoics, 7 Peterlee Place, Hebersham, NSW 2770; Novel Global Community Educational Foundation, Australia

**Keywords:** skin cancer, clinical trials, combination strategies, clinical implications, patient

## Abstract

Cutaneous Squamous Cell Carcinoma (cSCC) is a common and potentially fatal type of skin cancer that poses a significant threat to public health and has a high prevalence rate. Exposure to ultraviolet radiation on the skin surface increases the risk of cSCC, especially in those with genetic syndromes like xerodermapigmentosum and epidermolysis bullosa. Therefore, understanding the molecular pathogenesis of cSCC is critical for developing personalized treatment approaches that are effective in cSCC. This article provides a comprehensive overview of current knowledge of cSCC pathogenesis, emphasizing dysregulated signaling pathways and the significance of molecular profiling. Several limitations and challenges associated with conventional therapies, however, are identified, stressing the need for novel therapeutic strategies. The article further discusses molecular targets and therapeutic approaches, i.e., epidermal growth factor receptor inhibitors, hedgehog pathway inhibitors, and PI3K/AKT/mTOR pathway inhibitors, as well as emerging molecular targets and therapeutic agents. The manuscript explores resistance mechanisms to molecularly targeted therapies and proposes methods to overcome them, including combination strategies, rational design, and optimization. The clinical implications and patient outcomes of molecular-targeted treatments are assessed, including response rates and survival outcomes. The management of adverse events and toxicities in molecular-targeted therapies is crucial and requires careful monitoring and control. The paper further discusses future directions for therapeutic advancement and research in this area, as well as the difficulties and constraints associated with conventional therapies.

## Introduction

Cutaneous Squamous Cell Carcinoma (cSCC) is a common type of skin cancer that begins in the outermost layer of squamous cells. It poses a significant threat to public health due to its high prevalence and potential for aggressive behavior (Farberg et al., 2022[58]). Typically, cSCC develops in areas that receive ultraviolet radiation, i.e., the face, scalp, ears, and hands. The main cause of risk for cSCC is cumulative sun exposure over a lifetime (Farberg et al., 2022[58]). Ultraviolet radiation from sunlight or artificially generated radiation, like tanning beds, damages DNA in skin cells, causing genetic mutations that promote the growth of cSCC (Pillai et al., 2023[141]). Fair-skinned individuals with diminished melanin protection and those with a history of sunburns are especially susceptible (Dzwierzynski, 2021[52]). Furthermore, genetic syndromes, i.e., xerodermapigmentosum and epidermolysis bullosa, may enhance the risk of being diagnosed with cSCC (Winge et al., 2023[184]). Due to impaired immune surveillance against cancerous cells, immunocompromised individuals, like organ transplant recipients or those infected with the human immunodeficiency virus, have an increased susceptibility to cSCC.

**Figure 1:**
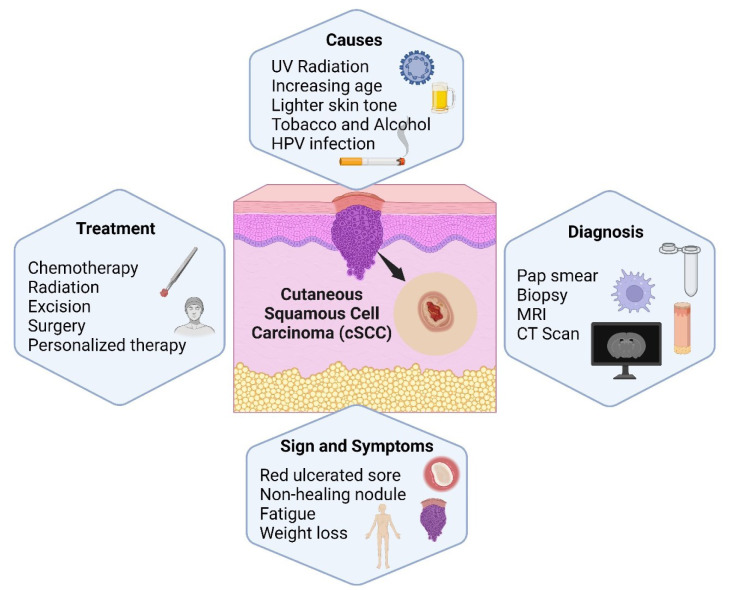
Overview on the causes, diagnosis, signs, and symptoms and the treatment approach used for cSCC. Figure created with BioRender.

The clinical symptoms of cSCC may contrast, but it usually appears as a scaly, firm, or crusty skin lesion. The lesion may look like a wart or an open wound and has the potential to grow rapidly, leading to symptoms including pain, tenderness, and bleeding. While cSCC is most commonly found in areas exposed to the sun, it can also develop in non-sun-exposed areas or be triggered by pre-existing skin conditions like actinic keratosis. Therefore, histopathological analysis is required for a conclusive diagnosis (Hult et al., 2020[84]). A microscopic examination reveals atypical squamous cells with varying degrees of differentiation that form nests or cords. The aggressiveness of cSCC can vary, with some tumors remaining localized and others exhibiting invasive behavior, which may result in metastasis and regional lymph node involvement (Brancaccio et al., 2021[31]). The choice of treatment depends on the variables, i.e., tumor size, location, invasion depth, and the presence of metastases. The detection and treatment of cSCC as soon as possible are crucial for a favorable outcome. In addition, surgical excision, cryosurgery, radiation therapy, topical therapies, and, in some cases, systemic therapies are available as treatment options for cSCC (Elleson et al., 2022[56]). Figure 1(Fig. 1) presents the causes, diagnosis, sign and symptoms related with cSCC.

In recent years, molecular targeted therapies have emerged as a promising treatment option for cSCC, giving patients new hope. The ability to specifically target the molecular alterations and signaling pathways that drive tumor growth and survival is one of the chief benefits of molecular targeted therapies in the treatment of cSCC (Chen et al., 2022[38]). Various molecular targeted therapies aim to inhibit or block certain molecules or signaling pathways that are vital to the growth and progression of cSCC, in contrast with traditional chemotherapy, which can affect both cancerous and healthy cells (Kurmi et al., 2020[105]). Thus, these therapies have the potential to result in more effective and less toxic treatments. Several molecular targets have been identified in cSCC, including the epidermal growth factor receptor pathway, the hedgehog pathway, and the mitogen-activated protein kinase pathway (Galambus and Tsai, 2023[66]). In clinical trials, drugs that target these pathways have shown promising results. Cetuximab and erlotinib are effective in treating advanced cSCC by inhibiting the activity of the epidermal growth factor receptor, which is often overexpressed in cSCC tumors (Chang et al., 2023[37]). Since the hedgehog pathway is crucial to the progression of cSCC, inhibitors of this pathway, i.e., vismodegib and sonidegib, have shown activity in both locally advanced and metastatic disease (Ascierto and Schadendorf, 2022[15]).

In addition, molecular targeted therapies offer the possibility of personalized medicine in the therapeutic management of cSCC. By analyzing the genetic and molecular characteristics of individual tumors, oncologists can identify specific molecular targets and create individualized treatment plans. This strategy has great potential for optimizing treatment outcomes and minimizing side effects (Cohen and Kurzrock, 2022[42]; Joshi, 2023[92]). Even though significant progress has been made in the field of molecular-targeted therapies for cSCC, obstacles still exist (Ghosh et al., 2022[72]). Over time, resistance to targeted therapies may develop, resulting in disease progression. In addition, not all cSCC tumors contain the same molecular alterations, which makes it imperative to identify the most pertinent targets for each patient.

This paper seeks to provide an in-depth review of molecular aspects, the current treatment landscape, molecular targets and therapeutic strategies, clinical trials and recent developments, resistance mechanisms and combination strategies, clinical implications, and patient outcomes, as well as safety, toxicity, and management approaches in the context of cSCC. The objective of this paper is to emphasize the value of comprehending the molecular pathogenesis of cSCC, the potential of molecular targeted therapies in improving patient outcomes, and molecular profiling for personalized treatment approaches. The paper also discusses future directions for therapeutic advancement and research in this area, as well as the difficulties and constraints associated with conventional therapies.

## Molecular Pathogenesis of cSCC

### Overview of signaling pathways dysregulated in cSCC

The dysregulation of signaling pathways is crucial to the development and progression of cSCC, contributing to its aggressiveness and metastatic potential. In cSCC, important pathways involved in cell proliferation, apoptosis, angiogenesis, and immune evasion are dysregulated. In cSCC, the epidermal growth factor receptor pathway is one of the signaling pathways that are most frequently aberrantly regulated. The epidermal growth factor receptor is overexpressed in a significant proportion of cSCC cases, which leads to increased cell proliferation and survival (London and Gallo, 2020[121]). The activation of epidermal growth factor receptors triggers signaling cascades, including MAPK and PI3K/ AKT pathways, that promote cell growth and suppress apoptosis (Chen et al., 2022[38]). When epidermal growth factor receptor (EGFR) signaling is out of whack in cSCC, cells multiply out of control and become resistant to apoptosis. This helps the tumor grow and spread (Winge et al., 2023[184]).

The Wnt/β-catenin signaling pathway is another dysregulated pathway in cSCC. This pathway is frequently activated abnormally by mutations in the β-catenin gene (CTNNB1), resulting in the accumulation of β-catenin in the nucleus, where it interacts with transcription factors to stimulate the expression of target genes involved in cell proliferation and survival (Liu et al., 2022[119]). The dysregulation of the Wnt/β-catenin pathway in cSCC contributes to tumor growth and invasiveness by promoting cell proliferation and inhibiting differentiation (Liu et al., 2022[119]). The cSCC exhibits alterations in angiogenesis-related signaling pathways and the intrinsic dysregulation of cell growth and survival pathways. Vascular endothelial growth factor and its receptors are upregulated in cSCC, promoting the development of fresh blood vessels, which aid tumor growth and metastasis (Elebiyo et al., 2022[54]). In cSCC, aberrant vascular endothelial growth factor signaling contributes to the formation of a pro-angiogenic microenvironment, which enables sustained tumor growth and invasion (Elebiyo et al., 2022[54]).

**Figure 2:**
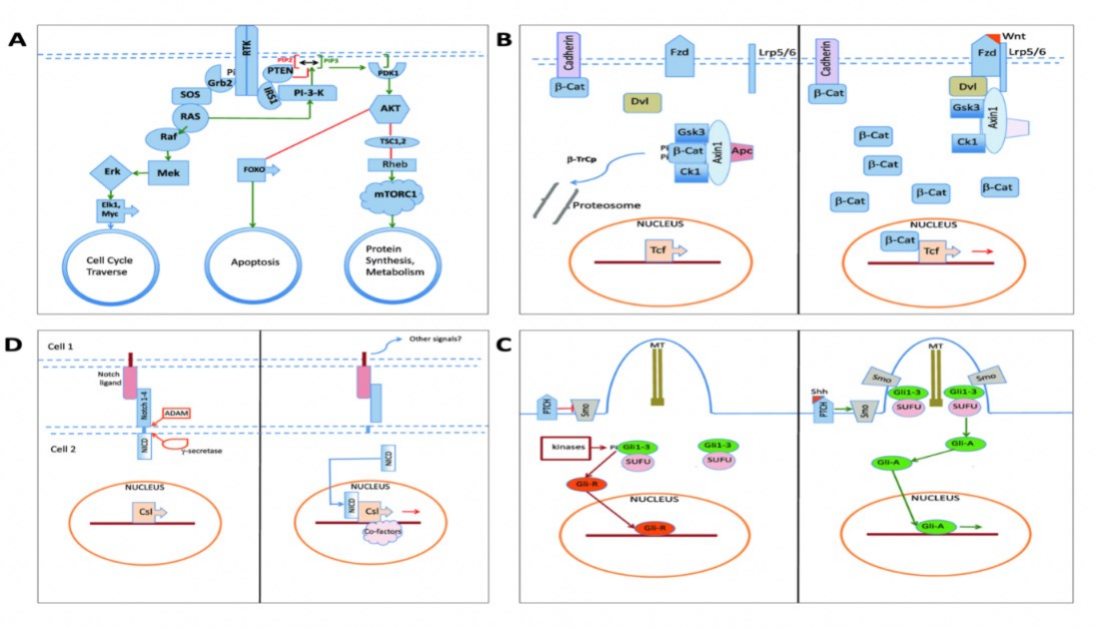
Various signaling pathways involved in the pathogenesis and therapeutic strategies of cSCC. (A) RAS signaling pathway, there are two key pathways involved: the MAP kinase pathway and the PI3K pathway. These regulate the cell cycle, death of cells, cell metabolism, and the production of proteins. Green arrows represent activation, whereas red lines represent inhibition. (B) The route of Wnt signaling. Catenin, a critical component of the Wnt pathway, has two roles. It functions as a transcriptional activator in the nucleus, regulating development of cell, as well as a component of cell adhesion junctions. (C) The Hedgehog route. The reaction of hedgehog ligands with the receptors in the membrane. (D) Notch Pathway. Cell-to-cell contact is required for Notch signaling. Notch ligands on a neighboring cell bind to Notch, leading it to undergo degradation by two proteases. This results in the release of the Notch cytoplasmic domain, which then migrates to the nucleus to communicate with transcription factors, reproduced with permission from reference (Juliano, 2020).

The dysregulation and signaling pathways of cSCC also have implications for immune evasion. The tumor microenvironment of cSCC frequently exhibits immunosuppression, with the presence of regulatory T cells (Tregs) and myeloid-derived suppressor cells (Haist et al., 2021[76]). The activation of immunosuppressive pathways, such as the transforming growth factor beta pathway, leads to the recruitment and functional impairment of immune cells, allowing tumor cells to evade immune surveillance and promoting tumor progression. One of the considerable changes in cSCC emerges at the molecular level, where the RAS-RAF-MEK-ERK pathway is activated. This pathway plays a crucial role in regulating cell growth and survival. In cSCC, mutations in the RAS or RAF genes can lead to the constant activation of the pathway, which promotes uncontrolled cell division and tumor growth. Therefore, the abnormal activation of the RAS-RAF-MEK-ERK pathway in cSCC makes it a likely therapeutic target (Fania et al., 2021[57]). In addition, alteration in cSCC involves inactivating tumor suppressor genes, like p53 and p16INK4a. The p53 protein acts as a guardian of the genome, regulating cell cycle progression, DNA repair, and cell death. In cSCC, mutation or loss of p53 function disrupts these critical cellular processes, allowing the accumulation of genetic mutations and promoting tumor development. Similarly, the loss of p16INK4a function, which normally regulates the cell cycle, can lead to uncontrolled cell proliferation in cSCC (Marei et al., 2021[124]). Figure 2(Fig. 2) (Reference in Figure 2: Juliano, 2020[93]) illustrates the signaling pathways involved in the pathogenesis and therapeutic strategies of cSCC.

Furthermore, cSCC is characterized by dysregulated cell cycle control. The cyclin-dependent kinase pathway, which regulates the progression of the cell cycle, is frequently disrupted in cSCC. Cyclins, cyclin-dependent kinases, and their inhibitors, such as p16INK4a, are altered in cSCC, resulting in uncontrolled cell cycle progression and increased proliferation (Juliano, 2020[93]). Targeting the abnormal development of cells could be an intriguing treatment option for cSCC tumor growth. Understanding the dysregulated signaling pathways in cSCC is essential for the development of targeted therapies and enhanced treatment (Luo et al., 2022[123]). Preclinical and clinical studies indicate that therapeutic interventions targeting key components of these pathways, such as epidermal growth factor receptor inhibitors, Wnt inhibitors, and antiangiogenic agents, hold promise (Singh et al., 2023[160]). By specifically targeting dysregulated signaling pathways, it may be possible to stop cSCC cells from growing and surviving. This could lead to more effective treatments and better outcomes for patients (Lazar et al., 2020[111]).

### Importance of molecular profiling for personalized treatment approaches

Molecular profiling is critical in personalized treatment approaches, particularly in cancer, including cSCC. Perhaps the most prevalent type of skin cancer is cSCC, and understanding its molecular pathogenesis is critical for developing targeted therapies and personalized treatment strategies (Lazo de la Vega et al., 2020[112]). Molecular profiling is the process of analysing an individual's tumor's genetic and molecular characteristics in order to identify specific alterations or biomarkers that can guide treatment decisions (Pishvaian et al., 2020[142]). Several healthcare providers can gain valuable insights into the underlying mechanisms driving cancer growth and progression by examining a tumor's molecular profile. This information can then be used to personalize treatment plans for each patient, enhancing the probability of a favorable therapeutic response (Lim et al., 2023[117]).

In cSCC, molecular profiling can aid in the identification of various molecular alterations that trigger the growth and advancement of the disease (Jones et al., 2021[91]). In cSCC, for example, studies have found mutations in genes like TP53, NOTCH1, NOTCH2, CDKN2A, and HRAS (Lee et al., 2022[115]). Understanding the presence and significance of these genetic alterations can aid in prognosis and treatment decisions. One of the major benefits of molecular profiling is its ability to identify potential therapeutic targets (Pishvaianet al.,2020[142]). Also, by looking at the molecular features of cSCC tumors, researchers can find specific genetic changes or signaling pathways that can be targeted by current drugs or therapies. For instance, cSCC with activating epidermal growth factor receptor mutations or overexpression can be treated with inhibitors of the epidermal growth factor receptor and its downstream signaling pathways (London and Gallo, 2020[121]). Furthermore, molecular profiling can help predict how a specific treatment will affect an individual patient (Sicklick et al., 2019[157]). Clinicians can identify biomarkers associated with drug sensitivity or resistance by analyzing a tumor's molecular profile (Chakravarty and Solit, 2021[35]). The changes in genes involved in drug metabolism, DNA repair mechanisms, or cell cycle regulation, for example, can impact a patient's response to chemotherapy or targeted therapies (Chakravarty and Solit, 2021[35]).

In one recent study related with personalized treatment approaches, only the immunocompromised patients were considered because most of the treatment options for cSCC would not work with them (Jones et al., 2021[91]). In this study, biopsies were taken from seven patients and sent for testing. A personalized treatment was given to them based on the results of the testing. The most common mutation observed was that of Tp53; however, in one case, a rare ERBB3 mutation was detected. A personalized treatment that included the addition of lapatinib to nivolumabwas given for six months. The patient's condition stabilized without further deterioration, as reported. Even though the sample size used in this case study was small to comment on the broader scope of personalized medicines, the results obtained indicate that personalized cancer treatment is achievable in the age of quick genetic and molecular tumor diagnosis, provided we are prepared to research each patient and take appropriate action based on the information found.

Through molecular profiling techniques such as next-generation sequencing, driver mutations have been identified in cSCC (Arnhold et al., 2017[14]). For instance, cSCC tumors frequently contain TP53 gene alterations (Piipponen et al., 2021[140]). Using cutting-edge treatments like MDM2 blockers or p53 reactivators, which work to restore the TP53 protein's normal function and slow tumor growth, clinicians can now specifically target the mutant TP53 pathway (Osman et al., 2020[136]). At present, the researchers are also emphasizing the state of drugs which target p53 drugs currently being used for the treatment of head and neck squamous cell carcinoma (HNSCC) (De Bakker et al. 2022[47]). The study showed how numerous research studies have examined various p53 reactivators to provide a solution to the demand for novel treatment regimens in HNSCC because personalized medicine is an efficient and better way of therapy that must be improved before being applied clinically.

A further study conducted in the year 2016 showed the importance of molecular profiling for personalized treatment approaches. In this study, analysis of 735 individuals who had metastatic HNSCC was done using different technologies (DNA sequencing, copy number analysis, and protein translation) (Feldman et al., 2016[60]). The study resulted in epidermal growth factor receptor, which favors metastasis, being highly expressed in most individuals. Tp53 is the most altered, which is in line with what De Bakker et al. (2022[47]) reported, and there were many other genes that had different expression and mutation levels in different individuals. Targeting these genes specific to those individuals, along with standard therapy, can help provide a precise therapeutic option for the patient (Ciardiello et al., 2022[41]). This data provides a thorough cataloguing of genetic variations, protein manifestations, and other appropriate molecular modifications. The information encourages the use of substances now undergoing clinical studies (PD1/PDL1), combination therapies, or substances authorized to be utilized in other solid tumor types (MGMT, HER2). Numerous methods for molecular profiling offer a variety of therapeutic alternatives in addition to conventional care (Kurzrock et al., 2020[107]). This suggests a thorough molecular profiling strategy to improve HNSCC personalized therapeutic options (Feldman et al., 2016[60]). In another study the use of genetic profiling to direct personalized treatment for advanced cSCC has been investigated on tumor samples from individuals with advanced cSCC. The researchers thoroughly profiled the genome, and individualized treatment plans were created by matching the discovered genetic abnormalities with specific medicines. This study showed how molecular profiling might be used to pinpoint therapeutically relevant mutations and direct the choice of targeted therapies in advanced cSCC (Al-Rohil et al., 2016[6]).

Importantly, molecular profiling can help guide clinical trials and the advancement of novel cSCC therapies (Bonini et al., 2023[26]). Researchers can further identify specific molecular alterations in tumors to design targeted therapies or develop new drugs that selectively inhibit aberrant molecular pathways driving cancer (Qian et al., 2020[146]). By providing more effective and tailored therapies, this approach has the potential to improve treatment outcomes.

## Current Treatment Landscape

### Surgical intervention for cSCC

Surgical interventions are essential to treat cSCC, especially for early-stage or localized tumors (Boutros et al., 2021[29]). The goal of surgical treatment is to eliminate all cancerous cells while preserving as much healthy tissue as possible (DiCorpo et al., 2020[49]). The type and size of the tumor, as well as an individual's personal characteristics, can all influence surgical procedures (Cortes-Guiral et al., 2021[44]). Excision is the most common surgical intervention for cSCC (Newman et al., 2021[132]). This procedure entails removing the tumor along with a border of healthy tissue. The extent of the margin depends on the size, location, and aggressiveness of the tumor (Ahmed, 2020[1]). The extracted tissue is then sent for pathological analysis to confirm that all cancer cells have been eliminated. Excision is frequently performed under local anesthesia in an outpatient setting and is usually linked with a relatively low incidence of complications (Ooi et al., 2022[135]).

For cSCC tumors that are larger or more invasive, more extensive surgical procedures may be required (García-Foncillas et al., 2022[68]). Mohs micrographic surgery is a specialized procedure employed for treating cSCC, particularly in vulnerable or difficult areas like the head, neck, and hands (Mohs, 1989[128]). During Mohs surgery, the tumor is removed layer by layer, with each layer being examined under a microscope immediately after removal (Mohs, 1989[128]). This enables the surgeon to precisely map and eliminate all cancerous cells while sparing healthy tissue (Aslam and Aasi, 2019[17]). Mohs surgery has a high cure rate and minimizes the removal of non-cancerous tissue, which makes it particularly advantageous for tumors with ill-defined borders or those located in cosmetically sensitive areas (Asgari et al., 2012[16]). In cases where cSCC has spread to nearby lymph nodes, lymphadenectomy or lymph node dissection may be performed (Janković et al., 2021[87]). This involves surgically removing the affected lymph nodes to prevent the cancer from spreading. Concerning the extent of lymph node involvement, lymphadenectomy is frequently performed in conjunction with excision or Mohs surgery and may be followed by adjuvant therapies such as radiation or systemic treatment (Gauci et al., 2022[69]).

Alternative interventions may be considered when the tumor is not amenable to surgical excision or when the patient is not a good surgical candidate due to underlying health conditions (Szturz et al., 2020[170]). These techniques can include cryotherapy (freezing the tumor), curettage and electrodesiccation (scraping and burning the tumor), and laser therapy (Kash and Silapunt, 2020[97]). Typically, these treatments are reserved for smaller, superficial cSCC lesions or instances where surgery may be difficult (Firnhaber, 2020[61]).

In the current scenario, sentinel lymph node biopsy (SLNB) and its use in the management of high-risk cSCC are studied. Related with this, an analysis was performed on 26 advanced cSCC subjects who received biopsies. The dye technique, or a combination dye and radioisotope procedure, was used to do the biopsies. Overall, it gave a summary of the most recent research and recommendations for SLNB, including patient selection standards, technical considerations, and effects on prognosis and therapeutic options (Takahashi et al., 2014[171]). This study also showed that the biopsies contributed to determining if sentinel node metastases were present, assisted in the prompt identification and management of latent lymph node metastasis, and consequently assisted in predicting patient prognosis. These findings imply that in situations of cSCC, the size of the initial lesion serves as a diagnostic factor for the implementation of SNB (Stătescu et al., 2023[164]). It is further recommended to perform a sentinel lymph node biopsy in cases where the tumor thickness ranges from 2 mm to 5 mm (Kofler et al., 2021[101]; Takahashi et al., 2014[171]).

In the year 2021, empirical investigation has been done to access the results of MMS for cSCC. In a group of patients with cSCC, the researchers evaluated the cure rates, recurrence rates, and cosmetic effects of MMS. The objective of this study was to assess the efficacy and aesthetic benefits of MMS in the treatment of cSCC. It showed that MMS increased the efficacy of treatment and decreased the additional therapies required post-operation (Roberson et al., 2021[149]). Several significant risks and complications associated with surgical intervention for cSCC include infection, bleeding, poor wound healing, and cosmetic issues (Cozma et al., 2023[45]). Furthermore, patients must discuss the probable benefits, risks, and anticipated outcomes with their healthcare providers in order to make informed decisions regarding the most appropriate surgical approach (Albahri et al., 2023[3]).

### Radiation therapy in cSCC management

Radiation therapy is an important treatment modality for cSCC. Based on the characteristics of the tumor and the health of the patient as a whole, it is frequently used as a primary treatment or in conjunction with surgery or chemotherapy (Stratigos et al., 2020[167]). A thorough evaluation is performed before beginning radiation therapy to determine the best treatment plan (Borgonovo et al., 2022[27]). This includes a thorough evaluation of the tumor's size, location, invasion depth, and involvement of nearby lymph nodes. To aid in treatment planning, imaging techniques like computed tomography scans or magnetic resonance imaging may be used (Thapa et al., 2021[173]).

The most common type of radiation therapy for cSCC is external beam radiation therapy (Bellia et al., 2019[22]). It entails precisely targeting the tumor site with high-energy radiation delivered from a machine outside the body (Bellia et al., 2019[22]). External beam radiation therapy is typically administered in daily fractions over several weeks to allow normal cells to heal between treatments (Baskar et al., 2012[21]). External beam radiation therapy aims to destroy or inhibit the growth of cancer cells while causing the least amount of damage to healthy surrounding tissues (Anand et al., 2021[11]). Another type of radiation therapy that may be used for cSCC that is limited to the superficial layers of the skin, such as early-stage tumors, is superficial radiation therapy (Yu et al., 2022[190]). Superficial radiation therapy uses low-energy radiation beams that penetrate the skin only a short distance, making it ideal for treating surface lesions (Pashazadeh et al., 2019[137]). This technique is especially effective for smaller, well-defined cSCC, providing excellent cosmetic results while preserving healthy tissue (Quazi et al., 2020[147]).

Radiation therapy may be used in conjunction with surgery or chemotherapy in some cases (Bennardo et al., 2021[24]). This multimodal approach is frequently recommended for advanced cases of cSCC or when the tumor has invaded nearby structures or lymph nodes. Radiation therapy can be used before or after surgery to decrease the size of the tumor, thus making it easier to remove, as well as to kill any cancer cells that remain and lower the risk of recurrence. The study related with evaluation of the efficiency of high-dose-rate electronic brachytherapy (HDR-EBT) has been done which acted as an alternate treatment option for patients that cannot undergo surgery. The tumor was confirmed to be a malignant basal cell or squamous cell carcinoma. A 50 Gy dose was given, and the viability of the treatment, side effects, esthetic look, and area-specific recurrence were evaluated. The results showed that the effectiveness and patient friendliness of HDR-EBT for basal and squamous cell carcinomas were encouraging and that all treated locations had shown favorable outcomes in subsequent monitoring. However, certain precautions are recommended to avoid side effects (Goyal et al., 2021[75]). In another study, the authors evaluated the potential long-term outcomes and predictive factors of definitive radiotherapy for early-stage glottic laryngeal squamous cell carcinoma (Lim et al., 2023[117]). They analyzed a cohort of patients who received radiotherapy as the primary treatment and assessed treatment outcomes, including local control rates and survival rates. The 66 Gy dose was the median, and further complications were assessed. The results showed that in early-stage glottic laryngeal squamous cell carcinoma, definitive radiotherapy produced a significant response rate, a better ability to speak, and manageable side effects. Prognostic indicators included the T2 stage and anterior commissural involvement. However, it is also worth mentioning that to lower the likelihood of ipsilateral tumor resurgence, radiotherapy needs to be further optimized (Lim et al., 2023[117]).

Allen et al. examined the long-term effects of radiation therapy for the treatment of nasal cavity carcinomas in a retrospective study. The researchers assessed local control rates, survival outcomes, and treatment-related complications in various carcinomas, with squamous cell carcinoma constituting 66 % of the group. The relapse rate was significantly lower (28 %) than the survival rate (70-80 %) in 10 years. This long-term supervision reveals that for carcinomas, radiation therapy can offer reliable, lasting locoregional management and favorable survival results (Allen et al., 2008[4]).

Despite the fact that radiation therapy for cSCC tends to be acceptable, it can cause side effects (Muto and Pastore, 2021[130]). Acute side effects that are common include skin redness, itching, and dryness in the treated area. These issues typically disappear once treatment is finished. Long-term radiation therapy or higher radiation doses occasionally cause skin changes, fibrosis, or radiation dermatitis (Spałek, 2016[163]).

### Limitations and challenges associated with conventional therapies

Conventional therapies for cSCC have several limitations and challenges. While these therapies are effective in many cases, they are not without drawbacks and can present difficulties for both patients and healthcare providers. One drawback of traditional therapies for cSCC is the possibility of cosmetic disfigurement. Surgical excision, one of the most typical methods of managing cSCC, may result in significant tissue removal, resulting in visible scars or functional impairments, particularly when dealing with lesions in sensitive cosmetic areas like facial skin. This can affect the sense of self-worth and the value of the life of a patient, especially if the disfigurement is visible (Shen et al., 2021[155]). Other treatment modalities, such as radiation therapy or topical chemotherapeutic agents, may cause skin damage or pigmentation changes, affecting the patient's appearance even more (Yang et al., 2020[187]). Another difficulty is the possibility of recurrence. Although conventional therapy can effectively remove visible tumors, cSCC can recur or metastasize (Jennings and Schmults, 2010[88]). This is especially true in cases of high-risk cSCC, such as tumors with aggressive characteristics or those occurring in immunocompromised people. Recurrent or metastatic cSCC can be more difficult to detect and treat than primary tumors, necessitating additional interventions and potentially limiting treatment options (Cañueto et al., 2020[34]).

The location and size of the tumor also influence treatment modality selection. Some cSCC lesions may be anatomically difficult to locate or have extensive involvement. Due to the potential functional or cosmetic consequences, surgical removal of the tumor, which is typically the initial course of treatment, might not turn out to be feasible in these instances. Alternative treatments, like radiation therapy (Baskar et al., 2012[21]) or systemic therapy (Fitzgerald and Tsai, 2019[64]), may be considered in such cases. However, these alternatives may have their own limitations and side effects, complicating the treatment decision-making process even further. Furthermore, conventional therapies for cSCC may not be appropriate for some patients. For example, older people or those with significant comorbidities may be less tolerant of surgery or other aggressive interventions (Szturz et al., 2020[170]). Similarly, patients with compromised immune systems, like transplant recipients or people with certain autoimmune diseases, may have difficulty managing cSCC because of their impaired ability to mount an immune response to the tumor (Bossi and Lorini, 2021[28]). These factors may necessitate changes to treatment plans and the consideration of alternative approaches, each of which may have limitations or efficacy.

Through experimental studies, the limitations of high-risk cSCC were highlighted. It demonstrated that despite being widely used treatment modalities, surgery and radiation therapy may have limitations due to factors like tumor size, anatomical location, and involvement of crucial structures (Fitzgerald and O'Neill, 2012[63]). Even though surgery remains the major treatment option for cSCC, later stages of this disease, old age, and esthetic effects limit its use. The study also mentions in brief that new treatment options like molecular therapy and immunotherapy may help with the precise treatment of the disease in the future. The study reported on the challenges faced with conventional therapy for carcinomas of the maxillary sinus and evaluated the results of neoadjuvant intra-articular chemotherapy followed by radiation therapy and surgery. It showed that the treatment might work for controlling the growth of the tumor; however, local failure was still responsible for the greatest number of deaths (Kim et al., 2013[99]).

Various investigations have further revealed the challenges and restrictions of treating advanced cSCC (Salzmann et al., 2020[151]). A recent study examined treatment patterns and survival outcomes in a large patient group with advanced cSCC. Regardless of conventional therapies like surgery, radiation therapy, and systemic chemotherapy, there is an immense need to provide efficient treatment, the study revealed, as evident by the relatively low survival rates observed in the study population (Kramb et al., 2022[103]). In addition, immunotherapy, particularly anti-PD-1 treatment, is frequently mentioned in the literature as a potential future treatment option for cSCC (Amaral et al., 2019[9]; Kramb et al., 2022[103]). It is essential to emphasize that while these studies provide valuable insights, the specific limitations and challenges may vary on an individual basis and should be considered in the context of each patient's distinctive condition.

## Molecular Targets and Therapeutic Strategies

### Epidermal growth factor receptor inhibitors in cSCC treatment

Several inhibitors of the epidermal growth factor receptor have emerged as a promising therapy for cSCC (Karamouzis et al., 2007[96]). Although the majority of cases of cSCCare successfully treated with surgery, advanced or metastatic cSCC presents significant difficulties. In such cases, inhibitors of the epidermal growth factor receptor have demonstrated efficacy in both systemic and topical approaches to cSCC (Alter et al., 2013[7]). Cetuximab, an inhibitor of the systemic epidermal growth factor receptor, has shown clinical benefit in patients with advanced cSCC (Su et al., 2008[169]). These drugs work by inhibiting the activation of the epidermal growth factor receptor, a cell surface receptor that is essential for cancer cell growth and survival. These drugs can slow tumor growth and potentially induce tumor regression by inhibiting epidermal growth factor receptor signaling (Montaudié et al., 2020[129]). Studies have shown that cetuximab can improve response rates and survival rates for people with advanced cSCC who have not had surgery or radiation therapy to cure the cancer. This is true whether the drug is used alone or with chemotherapy (Kurosaki et al., 2021[106]).

Besides systemic therapy, topical epidermal growth factor receptor inhibitors are useful in treating cSCC (Inoue et al., 2020[86]). Erlotinib, an epidermal growth factor receptor inhibitor that is available as a topical cream, is one notable example (Gold et al., 2018[73]). Erlotinib inhibits epidermal growth factor receptor signaling locally when applied directly to cSCC lesions, targeting cancer cells while minimizing systemic exposure. Clinical trials have shown that topical erlotinib can reduce tumor size and improve clinical outcomes in patients with cSCC, particularly when surgical intervention is not feasible or desirable (Inoue et al., 2020[86]). While epidermal growth factor receptor inhibitors have been shown to be effective in treating cSCC, it is critical to consider potential side effects and resistance mechanisms. Skin rashes (Inoue et al., 2020[86]) and diarrhea (Hirsh et al., 2014[81]) are two common side effects of systemic epidermal growth factor receptor inhibitors that can be treated with supportive care. Furthermore, epidermal growth factor receptor inhibitors can develop acquired resistance over time, limiting their long-term effectiveness (Viloria-Petit et al., 2001[181]). Ongoing research is being conducted to identify strategies for overcoming resistance and improving patient outcomes.

Retrospective research involving 58 patients revealed the safety of cetuximab monotherapy. It was elucidated that use of cetuximab monotherapy is safe, especially in patients over 65. The objective response rate was good throughout the three months. This study shows that cetuximab is both reliable and successful for treating individuals with advanced cSCC, especially those who are old. These results show that cetuximab is an interesting drug that should be tested in new combinations, especially with immunotherapies like anti-PD-1 drugs (Montaudié et al., 2020[129]).

Another set of experiment was done to evaluate the effectiveness of EGFR inhibitor gefitinib in individuals suffering from cSCC who weren't candidates for curative treatment such as surgery or radiation. This was a phase II trial in which gefitinib was given orally to forty individuals until the disease progressed or the side effects became unbearable. The predetermined objective rate of response was 20 %, and the overall rate of response was 16 %. Gefitinib demonstrated sufficient action in incurable cSCC, and its unfavorable side effects were not particularly severe (Fania et al., 2021[57]; William et al., 2017[183]).

Research carried out with an aim to evaluate the effectiveness of erlotinib in the management of recurring or aggressive cSCC patients that were unresponsive to conventional treatments. The study assessed tumor response rates, progression-free survival, and adverse events in the treated patients. 150 mg of erlotinib was given daily. No expected toxicities were observed, and 10 % of the 29 patients responded to treatment. It was found that erlotinib was viable in conjunction with predicted toxicity for many individuals with recurrent cSCC. The findings suggested that erlotinib may have a beneficial effect in a subset of patients with advanced cSCC who have failed conventional therapy (Gold et al., 2018[73]). These studies demonstrate the effectiveness of EGFR inhibitors, such as cetuximab and erlotinib, in treating cSCC. They highlight the potential effectiveness of these targeted therapies in managing advanced, refractory, or metastatic cSCC cases.

### Hedgehog pathway inhibitors and their role in targeted therapy

As a promising class of targeted therapies for cSCC treatment, hedgehog pathway inhibitors have emerged (Harwood et al., 2005[77]). The hedgehog signaling pathway is crucial for tissue development, cell differentiation, and cell proliferation. Nevertheless, cSCC development and progression have been associated with aberrant activation of this pathway (Bai et al., 2018[19]). In this type of skin cancer, inhibiting the hedgehog pathway has become an attractive strategy for targeted therapy (Nguyen and Cho, 2022[133]). Smoothened protein constitutes one of the most essential hedgehog pathway components. Smoothened protein is a molecular switch that transmits signals further along the pathway (Zhang et al., 2021[192]). Inhibitors of the hedgehog signaling pathway function by selectively targeting and inhibiting the activity of the smoothened proteins, thereby interrupting the aberrant signaling cascade associated with cSCC. This inhibition prevents the activation of downstream effectors, which ultimately inhibits the growth and progression of tumors (Nguyen and Cho, 2022[133]). Several hedgehog pathway inhibitors have been developed and evaluated in clinical trials for the management of cSCC. Typically, vismodegib and sonidegib, which are FDA-approved inhibitors for the management of advanced or metastatic cSCC (Leavitt et al., 2019[113]), have shown remarkable efficacy in reducing tumor size and improving overall response rates in patients who are unfit for surgery or radiation therapy (Villani et al., 2022[180]).

For patients with locally advanced cSCC, where surgery or radiation therapy may be too risky or too invasive, focusing on the hedgehog pathway has shown particularly promising results (Migden et al., 2018[126]). Inhibitors of the hedgehog pathway provide a non-invasive treatment option that effectively controls tumor growth and prevents disease progression in these instances. In addition, they have demonstrated promise as neoadjuvant therapies, which can be administered before surgery to reduce tumor size and facilitate complete resection (Nguyen and Cho, 2022[133]).

Hedgehog pathway inhibitors are not without limitations, despite their success (Chmiel et al., 2022[39]). Over time, resistance to these inhibitors can develop, resulting in disease relapse or progression (Kuczynski et al., 2013[104]). This highlights the need for continued research and the development of combination therapies that target multiple signaling pathways to circumvent resistance mechanisms. Moreover, the adverse effects of hedgehog pathway inhibitors, like muscle spasms, hair loss, and altered taste sensation, can be problematic for patients and necessitate close monitoring and management (Lacouture et al., 2016[108]).

Recently case studies were done to evaluate the synergistic effect of cemiplimab and sonidegib for basal and cSCC. It was observed that nonresponsiveness of preauricular basal cell carcinoma towards treatment were given cemiplimab and sonidegib, which allowed for a full response while retaining the anti-PD-1 effect on cSCC (Colombo et al., 2023[43]). In another case study, cemiplimab was introduced to combat a nodal recurrence of cSCC after administration of sonidegib for retro-auricular basal cell carcinoma, and both tumors completely responded. Regardless of the individual's advanced age, they were able to keep receiving their comprehensive treatment for approximately 3 years in the second case and nearly 2 years in the first case, respectively.

In another study, the safety and effectiveness of vismodegib was accessed. In the phase 2 multicenter trial, researchers studied the overall response rate, duration of response, progression-free survival, and adverse events in the treated patients. After administration of the drug, there was an excision of the lesion. Three different cohorts were evaluated, and there were various effects, like muscle spam, that were commonly observed. The results did not fulfil the goals for efficacy since the elimination rate was not more than 50 % in all cohorts. Continuous dosing showed better results, and Vismodegib-related adverse effects were reversible after therapy (Sofen et al., 2015[161]). In another investigation spread across different regions of the world, the subjects obtained 150 mg of vismodegib via ingestion every day until their illness progressed or the side effects became unbearable. After nine months, the initial evaluation was carried out. The main finding of the investigation revealed that vismodegib significantly reduced malignancies in 43 % of the individuals and 30 % of participants with invasive basal cell carcinoma. According to another evaluation, the average duration of progression-free survival (PFS) among individuals with metastatic and locally progressed tumors was 9.5 months. Additionally, the clinical benefit rate was 75 %, and it was observed that vismodegib reduced tumor size, treated visible lesions, or stopped their growth for longer than six months. The secondary endpoint was 60 % and 46 % for local and metastatic tumors, respectively.

### PI3K/AKT/mTOR pathway inhibitors for cSCC management

PI3K/AKT/mTOR pathway inhibitors have emerged as a promising class of targeted therapies for the treatment of cSCC (Jiang et al., 2020[89]; Ye et al., 2023[188]). The PI3K/AKT/ mTOR signaling pathway is an important intracellular pathway that regulates the growth of cells, survival, and metabolism (Ebrahimi et al., 2022[53]; Nepstad et al., 2020[131]). This pathway's dysregulation has been linked to the occurrence and development of cSCC, which makes it a desirable target for medicinal therapy. The PI3K/AKT/mTOR pathway inhibitors work by targeting specific components of the pathway to interrupt the signaling cascade and inhibit tumor growth (Mercurio et al., 2021[125]). Phosphoinositide 3-kinases are enzymes that play a vital role in activating downstream signaling through the pathway (Davé and Uversky, 2023[46]). Inhibitors targeting phosphoinositide 3-kinases block their activity and prevent the generation of secondary messengers that drive AKT and mTOR activation (He et al., 2021[78]). AKT is a protein kinase that promotes cell survival and proliferation, while mTOR regulates protein synthesis and cell growth. The inhibitors effectively disrupt aberrant signaling and halt the growth of cSCC by inhibiting these key components in the pathway (Liu et al., 2009[120]).

While PI3K/AKT/mTOR pathway inhibitors offer significant potential, they also come with limitations and potential side effects (Khan et al., 2019[98]). Off-target effects and dose-dependent toxicities have been reported, including skin rash, mucositis, hyperglycemia, and gastrointestinal disturbances (Shyam Sunder et al., 2023[156]). Close monitoring and management of side effects are necessary in order to safeguard patients and improve the results of treatment.

A phase II, multicenter, single-arm, open-label study performed in which researcher evaluated the administration of the PI3K inhibitor Buparlisib (BKM120). Participants first got one daily dose of 100 mg BKM120 tablets. Participants initially received one 100 mg BKM120 tablet daily. The primary endpoints were progression-free survival at 8 weeks of treatment (for high-grade strata) or 12 weeks of treatment (for low-grade strata), overall response rate, and safety. Due to the high toxicity rates observed at 100 mg, the dose was decreased to 60 mg; however, the toxicity rates remained elevated (Heudel et al., 2017[79]). Several PI3K/AKT/ mTOR pathway inhibitors for the treatment of cSCC have been developed and assessed in preclinical and clinical studies (Alzahrani, 2019[8]; De Kort et al., 2021[48]). These inhibitors demonstrate promising results, especially in advanced or metastatic cSCC cases where surgical or radiation options may not be feasible. It has been found that inhibiting the PI3K/AKT/mTOR pathway induces apoptosis in cSCC cells, inhibits tumor cell proliferation (Ghafouri-Fard et al., 2022[71]), and decreases tumor angiogenesis (new blood vessel formation to promote tumor growth) (Perrotte et al., 1999[139]).

Using PI3K/AKT/mTOR pathway inhibitors along with other treatments like immunotherapy or chemotherapy is something that is being looked into right now (Zhu et al., 2022[194]). Combinatorial approaches try to make treatments work better by focusing on more than one pathway at the same time or by using the synergistic effects of different types of treatments (Poon et al., 2021[144]). Combining different therapy strategies shows promise for improving outcomes and addressing resistance mechanisms that may develop with single-agent therapy. However, using BKM120 alone was associated with a poor safety profile and limited antitumor efficacy in these cancers. A clinical trial was terminated due to toxicity before the recruitment deadline. The trial exhibited high toxicity, and different adverse effects were observed (Heudel et al., 2017[79]).

In another study, it was showed the effectiveness of the mTOR inhibitor everolimus in people with recurrent or metastatic HNSCC because of the activation of PI3K, AKT, and mTOR. In this single-arm phase 2 trial, everolimus was given until toxicity levels increased. The primary endpoint was the clinical benefit rate (CBR). Secondary endpoints included PFS, overall survival, and analysis of tissue and serum biomarkers linked to the PIK3CA pathway. CBR was 28 %, and no objective response rate was seen. There were no PI3K-triggering changes found in the available tumor tissue. In individuals who had HNSCC that was recurrent or had spread to other organs, everolimus was ineffective as monotherapy. However, it should be noted that one constraint of the research was the small sample size of only seven people (Geiger et al., 2016[70]). At present, researchers are working on current evidence on targeting the PI3K/AKT/mTOR pathway in the treatment of cSCC. The study discussed the preclinical and clinical data available for PI3K/AKT/ mTOR inhibitors, highlighting their potential to inhibit tumor growth and promote cell death in cSCC. There is some information in this article about why and how PI3K/AKT/ mTOR pathway inhibitors might be used to treat cSCC (Mercurio et al., 2021[125]).

### Other emerging molecular targets and therapeutic agents

Besides the PI3K/AKT/mTOR pathway inhibitors, the Hedgehog pathway, and the epidermal growth factor receptor pathway, there are new molecular targets that are being looked at to treat cSCC (Chamcheu et al., 2019[36]). Two of these targets are the RAS-RAF-MEK-ERK pathway and the Wnt/β-catenin signaling pathway (Steelman et al., 2011[165]). Understanding the role of these pathways in cSCC development and identifying therapeutic agents that target them hold promise for improving treatment outcomes. The RAS-RAF-MEK-ERK signaling pathway is essential for the proliferation of cells, their survival, and differentiation. Mutations in the RAS and RAF genes, which are key components of this pathway, are frequently observed in cSCC (Su et al., 2012[168]). These mutations lead to constitutive activation of the pathway, which drives uncontrollable cell growth and tumor progression. As a result, targeting the RAS/RAF/MEK/ERK pathway as a possible approach to treatment has emerged. The effect of the RAS/RAF/MEK/ERK signaling pathway on the pathogenesis of cSCC was evaluated and in this evaluation, researchers evaluated how defects in the protein signaling pathway led to uncontrolled growth, immense cell proliferation, evasion of apoptosis, and cancer cell survival, which are like biomarkers of cSCC (Yang and Liu, 2017[186]). The researchers also mention that more research needs to be carried out on the RAS/RAF/ MEK/ERK signaling pathway to study its role in cSCC. Targeting the RAS/RAF/MEK/ERK signaling pathway has produced fresh perspectives and new therapeutic targets (Dillon et al., 2021[50]; Doghish et al., 2023[51]). One big problem that needs to be solved when trying to target the Ras/Raf/MEK/ERK pathway is the amount of cross-talk and negative feedback that happens on other pathways, such as the mTOR pathway (Yang and Liu, 2017[186]). FDA has approved multiple target inhibitors for cancer treatment. Inhibitors that target the Ras/Raf/ MEK/ERK pathway demonstrated a significant tumor suppressive effect (Song et al. 2023[162]). Drug resistance is a challenge even in targeted therapies. However, the use of RAF and MEK inhibitors has the distinct advantages of specific targeting, effective inhibition, and minimal side effects. RAF inhibitors, such as vemurafenib and dabrafenib, exhibit efficacy for the management of cSCC by activating BRAF mutations (Alqathama, 2020[5]). These inhibitors specifically target the mutated BRAF protein, inhibiting its activity and downstream signaling. By doing so, they help to normalize the aberrant signaling in the RAS/RAF/MEK/ERK pathway and impede tumor growth (Feichtenschlager et al., 2023[59]; Hofmann et al., 2021[82]).

Another emerging molecular target in cSCC is the Wnt/β-catenin signaling pathway (Yuan et al., 2021[191]). This pathway is essential for embryonic development and tissue homeostasis, but its dysregulation has been linked to several types of cancer, including cSCC (Hiremath et al., 2022[80]). Activation of the Wnt/β-catenin pathway leads to increased cell proliferation and survival, promoting tumor development and metastasis. In contemporary time, researchers are working to identify that how targeting the Wnt/β-catenin signaling pathway helps in tumor suppression and cell cycle arrest (Zhang and Wang, 2020[193]). The key components of the Wnt/β-catenin pathway include the Wnt ligand, the TCF/β-catenin complex, and the β-catenin destruction complex. Although the Wnt/β-catenin pathway is an optimistic approach, there are evidently off-targeting effects that need improvement. The combinatorial research also needs to be further advanced. Therapeutic agents targeting the Wnt/β-catenin pathway are being investigated in preclinical and early-phase clinical trials. These agents aim to inhibit the activity of vital components in the pathway, such as β-catenin or the Wnt ligands themselves, to restore normal signaling (Prosperi and Goss, 2010[145]). By blocking aberrant Wnt/β-catenin signaling, these agents may inhibit tumor development and improve treatment outcomes for cSCC.

It is important to note that targeted therapies for the RAS/RAF/MEK/ERK pathway and the Wnt/β-catenin signaling pathway are still in the early stages of development and clinical implementation. There are certain challenges that need to be addressed in order to achieve optimal therapeutic efficacy, such as the emergence of resistance mechanisms and potential toxicities. Combinatorial approaches that target multiple pathways at the same time or targeted therapies combined with other treatment methods, such as immunotherapy, may also show promise in the fight against cSCC. Studies have confirmed that the RAS/RAF/MEK/ERK signaling pathway can be used as inhibitors in combination with many existing therapies such as radiotherapy, chemotherapy, autophagy inhibitors and immune inhibitors. RAS/RAF/MEK/ERK pathway inhibitors, along with platinum-based chemotherapy, showed advantageous effects. Similarly, autophagy inhibition on molecular signaling pathways arrested cell growth and promoted tumor suppression. Immunotherapy, specifically anti-PD-1, in combination with MEK inhibitors, increased immune cell infiltration and reduced tumor progression (Song et al., 2023[162]).

## Clinical Trials and Upcoming Prospects for Remedial Progress

### Overview of clinical trials evaluating molecular targeted therapies in cSCC

Clinical trials evaluating molecular targeted therapies for cSCC have emerged as a crucial avenue for advancing treatment options and enhancing patient outcomes for this aggressive form of skin cancer (Al-Rohil et al., 2016[6]). The goal of these studies is to find out how safe, effective, and well-tolerated specific targeted therapies are that are meant to stop key molecular changes and signaling pathways that are linked to the start and growth of cSCC. The epidermal growth factor receptor is an important target in cSCC clinical trials. In advanced cSCC, inhibitors of the epidermal growth factor receptor, like cetuximab and panitumumab, have been intensively investigated in clinical trials (Yewale et al., 2013[189]). To assess objective response rates, progression-free survival, and overall survival, these trials typically employ single-arm or phase II designs. The results indicate varying degrees of success, with objective response rates ranging between 25 and 40 percent (Korn et al., 2008[102]). The emergence of resistance mechanisms and the occurrence of toxicities, i.e., skin rash and diarrhea, have highlighted the need for additional research and optimization of treatment regimens.

In cSCC clinical trials, the dysregulation of the hedgehog signaling pathway has also been a major focus. Vismodegib, an inhibitor of the hedgehog pathway, inhibits this pathway with promising results in locally advanced and metastatic cSCC (Sandhiya et al., 2013[152]). Randomized controlled trials or single-arm designs have been used in most clinical trials of vismodegib to look at objective response rates, duration of response, and side effects (Ladanie et al., 2019[109]). Approximately 30 % of objective response rates have been reported, demonstrating clinical benefit in a subset of patients. However, acquired resistance and adverse events, such as muscle cramps and alopecia, have been observed, necessitating additional research to optimize treatment strategies and reduce toxicity. One study talks about the special function of long non-coding RNA BBOX1 antisense RNA 1 (AS1) in squamous cell carcinoma cells and the underlying regulatory mechanism (Hu et al., 2023[83]). BBOX1-AS1 is abnormally upregulated. While BBOX1-AS1 overexpression had the opposite effects, BBOX1-AS1 depletion had inhibitory effects on cell proliferation and stemness. BBOX1-AS1 was also shown to stimulate the hedgehog signaling pathway. This pathway plays a vital role in maintaining tissue homeostasis and embryogenesis.

Other molecular targets beyond the epidermal growth factor receptor and hedgehog signaling have been investigated in clinical trials for cSCC. The PI3K/AKT/mTOR pathway, known to be essential for cell survival and proliferation, is a potential therapeutic target (Li et al., 2014[116]). In early-phase clinical trials for cSCC, inhibitors of this pathway, i.e., everolimus and temsirolimus, have demonstrated activity. To assess safety, tolerability, and preliminary efficacy, these trials commonly employ dose escalation or expansion cohorts (Bendell et al., 2015[23]). In addition, clinical trials have been conducted with angiogenesis inhibitors such as bevacizumab and sorafenib due to their ability to disrupt tumor blood supply (Al-Abd et al., 2017[2]).

Immunotherapy, particularly immune checkpoint inhibitors, is being recognized as a groundbreaking cancer treatment, including for cSCC (Humeau et al., 2021[85]). In clinical trials for advanced cSCC, antibodies targeting programed cell death protein 1 (PD-1) and its ligand (PD-L1) have shown promising results (Vaishampayan et al., 2023[178]). The PD-1 inhibitors pembrolizumab and cemiplimab have been given expedited approval by regulatory bodies to treat metastatic or locally advanced cSCC. Typically, these clinical trials employ multicenter, open-label designs with endpoints comprising the overall response rate, duration of response, and safety profiles. The results have demonstrated a substantial clinical benefit, resulting in the approval of these agents for specific indications. A study on targeting tumor-associated macrophages (TAM) for successful immunotherapy elucidates that TAM has gained a lot of importance in recent years (Truxova et al. 2023[174]). They comprise infiltrating immune cells and are heavily associated with tumor progression. Therefore, TAMs are notable targets for treating cancer. However, better research needs to be carried out for differentiating cancer, promoting TAM, and anticancer TAM. A combination of immunotherapy focusing on TAM and other molecular therapies would be a good approach.

### Future directions for therapeutic advancements

Several promising future directions are emerging in the field of therapeutic advances for cSCC as research and medical advancements continue to evolve. Targeted therapy is a major area of concentration. Currently, targeted therapies have revolutionized the management of different types of cancers, and efforts are underway to identify the molecular changes that drive the growth and progression of cSCC (Urruticoechea et al., 2010[177]). By identifying these key genetic and molecular abnormalities, scientists can create drugs that selectively target and inhibit the signaling pathways involved in the growth of cancer cells. This method holds great promise for personalized medicine as it enables the development of individualized treatment strategies based on genetic profiles.

Immunotherapy is another area of cSCC treatment that is undergoing rapid development. Immunotherapeutic strategies aim to stimulate the immune system to identify and destroy cancer cells. Recent advances in immune checkpoint inhibitors, such as anti-PD-1 and anti-CTLA-4 antibodies, have shown noteworthy efficacy in the treatment of advanced cSCC (Miller et al., 2017[127]). Current research focuses on the development of new immunotherapeutic agents, the investigation of combination therapies, and the research of predictive biomarkers to identify patients who are most likely to reap the benefits of these types of therapies.

The potential for early detection and accurate monitoring of cSCC is enhanced by advances in molecular diagnostics and imaging techniques. Liquid biopsy, a non-invasive technique for detecting tumor-specific genetic alterations in blood samples, is gaining popularity as a tool for observing disease recurrence and treatment response (Lone et al., 2022[122]). In addition, the development of novel imaging modalities, such as molecular imaging and optical coherence tomography, allows for improved visualization and characterization of cSCC lesions, thereby facilitating accurate diagnosis and treatment planning (Jindal et al., 2023[90]).

Minimally invasive techniques are gaining prominence in the field of surgical intervention. Mohs micrographic surgery, which entails the removal and examination of successively thinner layers of tissue until no cancer cells remain, offers superior cure rates while preserving healthy tissue (Campbell and Youker, 2011[33]). Additionally, advances in robotic surgery and image-guided interventions are likely to improve surgical outcomes by enhancing surgical precision and reducing surgical complications.

Moreover, the rapidly expanding field of nanomedicine has enormous potential for the treatment of cSCC. Nanoparticles can be designed to specifically target cancerous cells, provide therapeutic agents right to the tumor site, and overcome drug resistance (Chopra et al., 2021[40]). These nanoparticles can also be used for imaging, allowing for the monitoring of treatment response and tumor regression in real-time.

Additional research and clinical trials are required to validate the efficacy and safety of these therapeutic advancements for cSCC, despite their great promise. Collaboration between clinicians, researchers, and pharmaceutical companies will be crucial to the advancement of these fields. By continuing to investigate novel therapeutic approaches, personalized treatments, and cutting-edge technologies, we can hope to significantly improve the prognosis and quality of life of cSCC patients in the coming years.

## Resistance Mechanisms and Combination Strategies

### Mechanisms of resistance to molecular targeted therapies and approaches to overcome resistance

The effectiveness of molecular targeted therapies in the management of cSCC has been established. However, the development of resistance to these therapies remains a significant obstacle that limits their effectiveness over the long term. Improving outcomes in the management of cSCC requires a deeper understanding of the mechanisms of resistance and the development of countermeasures against them. The activation of alternative signaling pathways has emerged as a form of resistance. Cancer cells can circumvent the targeted pathway and activate parallel or compensatory pathways to promote cell survival and proliferation (Von Manstein et al., 2013[182]). Combination therapies that simultaneously target multiple pathways have shown promise for overcoming this resistance mechanism. By inhibiting multiple signaling pathways, the likelihood that cancer cells will find alternative survival routes is diminished. Efficacy-enhancing combinations of targeted therapies with distinct mechanisms of action or with conventional chemotherapy have been investigated (Elgundi et al., 2017[55]).

Another resistance mechanism involves genetic modifications to the target molecule. The targeted protein is susceptible to mutations that render it less susceptible to the inhibitory effects of the therapy (Silver and Bostian, 1993[158]). To overcome this type of resistance, it is possible to create inhibitors of the next generation that can effectively target mutant forms of the protein. By designing drugs that can bind to the mutant protein and inhibit its activity, it is possible to restore the efficacy of targeted therapies (Tsuruo et al., 2003[175]).The activation of compensatory signaling pathways is an additional mechanism of resistance. Cancer cells can adapt to targeted therapies by activating additional survival signaling pathways. Combination therapies containing inhibitors that target both the primary pathway and the compensatory pathway have demonstrated the potential to overcome this type of resistance (Takebe et al., 2015[172]). By simultaneously blocking multiple pathways, the adaptive response of cancer cells can be prevented.

The tumor microenvironment is essential for resistance development. By providing supportive signals and physical barriers, stromal cells, immune cells, and the extracellular matrix can play a role in therapy resistance. Targeting the tumor microenvironment, such as with immunotherapies or agents that modify the tumor microenvironment, has demonstrated promise for enhancing the efficacy of targeted therapies. By modifying the tumor's microenvironment, resistance-promoting conditions can be eliminated (Wu and Dai, 2017[185]). Several strategies to overcome resistance in cSCC include the use of adjuvant therapy and treatment sequencing in addition to combination therapies. Adjuvant therapies are used in conjunction with targeted therapies to improve their efficacy. Combining targeted therapies with radiation therapy, for instance, has improved cSCC treatment outcomes (Gold et al., 2009[74]). Treatment sequencing entails administering therapies in particular order to maximize their efficacy and delay the emergence of resistance. By strategically timing the administration of various treatments, it is possible to delay or prevent the development of resistance mechanisms. It is essential to recognize that overcoming resistance requires a tailored strategy. The development of predictive biomarkers can assist in the assessment of patients who are more likely to develop resistance, allowing for the development of individualized treatment plans (Schmidt et al., 2016[153]). In addition, continuous monitoring of treatment response and early detection of resistance is essential for adapting treatment protocols and implementing the most effective therapeutic interventions. 

### Rational design and optimization of combination strategies

Rational design and optimization of combination strategies have become increasingly important in both the prevention and treatment of cSCC. Combination approaches aim to target multiple pathways or biological processes simultaneously with the objective of enhancing effectiveness, overcoming resistance, and lowering toxicity (Sindhu et al., 2021[159]). To design and optimize these strategies, an in-depth knowledge of the biology underlying cSCC, the mechanisms of action of individual agents, and their potential synergistic effects is required.

To begin, rational combination strategy design entails identifying complementary mechanisms of action. This necessitates an in-depth examination of the molecular pathways and biological processes involved in the development and progression of cSCC. Researchers can select agents that act at various sites of the pathway or target distinct biological processes by identifying key signaling pathways, genetic alterations, or specific targets that regulate cSCC. Combining inhibitors targeting both the EGFR and the PI3K/AKT/mTOR pathways, for example, can effectively block multiple survival signals in cSCC cells (Freudlsperger et al., 2011[65]). Besides complementary mechanisms of action, rational design entails selecting agents with preclinical or clinical evidence of synergy. When two or more agents are combined, the combined therapeutic effect is greater than the sum of their individual effects. Various approaches, such as high-throughput screening and computational modeling, can aid in the identification of potential synergistic combinations (Zimmermann et al., 2007[195]). These methods consider agents' molecular profiles, pharmacokinetics, and known interactions with targets or pathways. Once potential synergistic combinations have been identified, additional experimental validation is required to confirm their efficacy.

The timing and dosing of the agents are also considered when optimizing combination strategies. Treatment sequencing, or the sequential administration of agents, can be optimized to exploit the vulnerabilities of cSCC cells at various stages. For example, a targeted therapy could be used to suppress cancer cell growth first, followed by immunotherapy to activate the immune system against the remaining cancer cells (Lee et al., 2016[114]). Furthermore, agent dosing schedules must be carefully considered to minimize toxicity while maximizing therapeutic efficacy. Based on preclinical and clinical data, this may entail adjusting the dosage, frequency, or duration of administration. Furthermore, potential drug-drug interactions and adverse effects should be considered when developing combination strategies. Some agents may interact with one another, either synergistically or antagonistically, altering pharmacokinetics or increasing toxicity. To ensure an optimal combination design, these interactions must be carefully evaluated (Uijtendaal et al., 2014[176]). In addition, the toxicity profiles of individual agents must be considered to avoid cumulative or synergistic toxic effects that could jeopardize patient safety and tolerability. To manage toxicity and maintain the balance between efficacy and safety, close monitoring and dose adjustments may be required.

Various preclinical models, such as cell lines or animal models of cSCC, can be used to test different combinations and dosage regimens to optimize combination strategies (Ruggeri et al., 2014[150]). Before proceeding to clinical trials, researchers can use these models to assess the efficacy, toxicity, and mechanisms of action of combination therapies. In addition, biomarkers and molecular profiling can be utilized to determine predicted markers of response to specific combinations, enabling the selection of patients more likely to benefit from the treatment (Ruggeri et al., 2014[150]).

## Clinical Implications and Patient Outcomes

### Evaluation of response rates and survival outcomes in patients treated with molecular targeted therapy

In recent years, several targeted medicines for the treatment of cSCC have received approval from the US Food and Drug Administration. These medications are permitted for use in patients with relapsed or resistant illnesses or as first-line therapy for those who cannot receive rigorous chemotherapy due to age, performance status, or concomitant conditions. Through clinical studies and off-label prescription, they are also being used frontline in patients of various ages and activity levels. The medication cemiplimab (Libtayo), used to treat advanced cSCC, has been authorized by the FDA. This is the first drug that the FDA has expressly authorized for use in advanced cSCC. Immunological checkpoint inhibitors, of which cemiplimab is a member, function by enhancing the body's immunological response to tumors. The FDA's approval covers patients with metastatic or locally advanced cSCC who are not candidates for surgery or radiation therapy. The FDA approved cemiplimab based on the results of two early-phase clinical studies that included 108 patients (33 with locally advanced cancer and 75 with metastatic disease) (Ogata and Tsuchida, 2019[134]). 13 out of 26 patients in one of the studies reacted to cemiplimab. In the second study, cemiplimab treatment resulted in tumor reduction or disappearance in 28 of 59 patients with metastatic illness. 16 of the 28 patients with metastatic illness who had responses lasted longer than six months, and 13 of these patients were still responding and receiving cemiplimab at the conclusion of the analysis.

The findings of a case report have revealed that cemiplimab drug was used for the treatment of a patient on dialysis. While cemiplimab use in dialysis patients has not been reported, other PD-1-inhibiting monoclonal antibodies (such as nivolumab, ipilimumab, and pembrolizumab) have been used effectively as frontline therapies. Because of their large molecular weight (between 143 and 148 kDa), these compounds continue to be effective in dialysis patients without requiring a change in dosage. However, cemiplimab, like other PD-1 inhibitors, may be safe and effective for use in patients on dialysis (De Bakker et al., 2022[47]). To gain more insights on the combinatorial effect of cemiplimab and effect of radiotherapy when used in combination with the cemiplimab drug, studies are being performed which revealed that many cancer patients are developing resistance to this therapy (Bailly-Caillé et al., 2023[20]). Therefore, a combination of radiotherapy and immune-responsive drugs was used. According to the results based on real-life studies, the combined response was long-term and consistent.

### Identification of predictive biomarkers for treatment response 

A biomarker is a molecule secreted by a cancer cell, or it is generated by the response of the human body to the cancer cells. They are useful in staging cancer, risk estimation, and finding the best suitable treatment. Cancer biomarkers are categorized according to their disease status and include predictive, diagnostic, and prognostic biomarkers. A prognostic biomarker predicts a cancer outcome like cause-specific survival, irrespective of therapy. Predictive biomarkers, which predict the benefit of a medical intervention and guide the development and delivery of tailored therapies, i.e., "the right drug to the right patients," are in high demand (Rivera et al., 2017[148]). To establish that a biomarker predicts the efficacy of an intervention, an assessment of the intervention against control treatment in persons with and without the biomarker is normally required in randomized trials. Although investigating solely biomarker-positive individuals would confirm the efficacy of a certain intervention, it does not indicate the role of the biomarker specifically. As a result, it is often reasonable to stratify patients in randomized trials based on the existence or absence of the biomarker (if dichotomous). Because demonstrating that individuals who are positive for a biomarker and receive investigational therapy have an improved outcome compared to those who get the same treatment but are negative for the biomarker does not prove that the biomarker is predictive. Differences in results linked to the biomarker may be owing to the biomarker's prognostic capabilities and may exist regardless of the therapy received. The higher variations in therapy and control in biomarker positive vs. biomarker negative groups are what establishes the biomarker as predictive (Kivisaari and Kähäri, 2013[100]).

Due to the lack of effective techniques to identify predictive biomarkers, many anticancer medications must be utilized to their full potential. Consequently, some medications are routinely administered to patients who will not benefit from them, resulting in unnecessary toxicity and expenses. In contrast, others will only be approved if the researchers can determine the specific patient population in which they are effective. Despite these challenges, only a few reliable predictive biomarkers have been developed and validated, which has led to changes in the standard practices of oncology (Perez-Gracia et al., 2017[138]).

It is cleary evident that circular RNAs are emerging as therapeutic targets in cSCC. Researchers have investigated the role of circRNA, circ_0070934, in cSCC. Through a study of An et al., it has been determined that circ_0070934 is highly expressed in cSCC and that increased expression of circ_0070934 correlates with tumor aggressiveness (An et al., 2019[10]). Circ_0070934 influences the expression of miR-1238 and miR-1247-5p to increase cSCC cell proliferation and invasion. Thereby, circ_0070934 can act as a potential predictive biomarker. Finding a predictive biomarker is a daunting task. Not all potential molecules turn into biomarkers. One such example is research carried out by where this group investigated the hypothesis that an increased number of EGFR gene copies acts as a predictive biomarker for squamous cell carcinoma. Dual-color FISH analysis was used as a method, and the results indicated that elevated levels of EGFR cannot be used as a predictive biomarker.

### Considerations for personalized treatment selection

**Figure 3:**
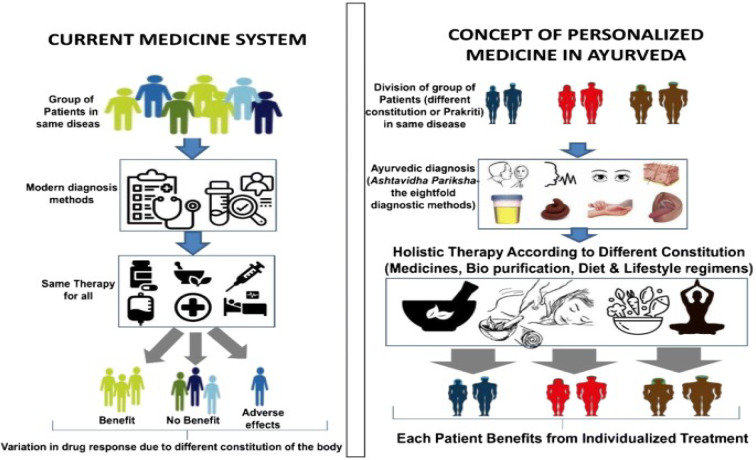
Current medicinal system and its lacunae in the treatment of various diseases in comparison with personalized medicine in Ayurveda and its efficacy for individual patients, reproduced with permission from Sharma and Prajapati (2020), Copyright 2020, Springer.

**Figure 4:**
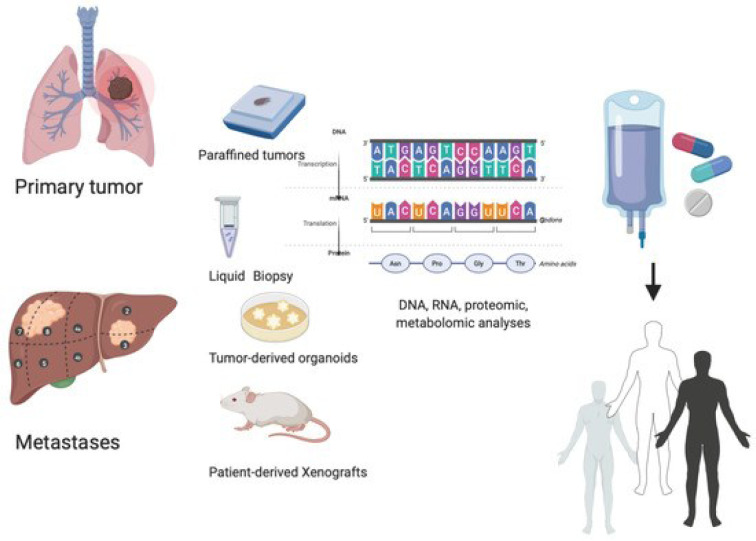
Personalized therapy using liquid biopsy, tumor-derived organoids, and tumor-derived xenografts based on genomic assessment of the original tumor or metastases, reproduced with permission from Gambardella et al. (2020)

Because of the wide diversity of patients with respect to sickness symptoms, performance level, and comorbidities, persons aged 60 to 75 must choose between intensive chemotherapy and specialized treatment. However, the effects of personalized therapy vs. intensive chemotherapy on survival and response rates have not been well studied. Patient staging and categorization are critical in determining the optimum therapy strategy. After completing an acceptable diagnosis, finding a suitable treatment is a critical step. The technique of therapy varies from person to person, but the essential objective remains the same: tumor excision and slowing progression. Over the last decade, it has become more obvious that no two individuals' malignancies are precisely the same, and hence how they respond to generic therapies like chemotherapy and radiation may vary. This standard approach to cancer therapy is unnecessarily simple, resulting in inefficient, costly therapies that subject patients to unwanted side effects. Precision and personalized medicine (PPM) is a more effective methodology positioned to transform this "one size fits all" approach. Figure 3(Fig. 3) (Reference in Figure 3: Sharma and Prajapati, 2020[154]) exhibits the comparative study related with current medicinal system and its lacunae in the treatment of various disease in comparison with personalized medicine. This point of view encourages the development of specialized cancer treatments that are based on measuring and manipulating important genetic and omics data about the patient, such as transcriptomics, metabolomics, and proteomics. Given the complexity and interpatient heterogeneity of cancer, it is evident that incorporating a PPM perspective into cancer research and treatment could result in significant gains in cancer combat. This has begun to be acknowledged in the current state of science and medicine through PPM studies, PPM products, and FDA support; however, there are a number of larger social barriers that must be discussed and overcome prior to PPM becoming fully integrated into established care. Genetic factors, autoimmune diseases, age, weight, stress level, smoking habit, level of alcohol consumption, type of food intake, presence or absence of diabetes, and cardiovascular diseases are the various factors to be taken into consideration while giving a personalized treatment (Avishai et al., 2017[18]). Figure 4(Fig. 4) (Reference in Figure 4: Gambardella et al., 2020[67]) presents the important aspects of personalized therapy based on genomic assessment of the original tumor or metastases.

The trend to utilize personalized therapy for squamous cell carcinoma is increasing, wherein it has been observed no significant shrink in the tumor size over short-term exposure to drugs and treatment. However, prolonged exposure had a positive response. The researchers also mentioned that there is a possibility of drug resistance in the near future, so the mechanism of resistance should also be studied individually as well as in a combined treatment (Karamboulas et al., 2018[95]). Another study demonstrates that molecular targeted therapies are not effective for all patients; however, when some patients are given personalized medicines, they were effective and had a positive impact (Arnedos et al., 2014[13]). Personalized medicine provides a specialized combination which even includes the mode of drug delivery. It also helps in avoiding toxicity. In addition, personalized treatment used was immunotherapy with PDL1, PD1, ALK inhibitor crizotinib. 

## Safety, Toxicity and Management Strategies

### Adverse events associated with molecular targeted therapies 

Molecular targeted therapies have emerged as a promising treatment option to prevent cSCC, a prevalent form of skin cancer. However, while these therapies offer several advantages, it is crucial to be aware of their potential side effects during usage. The adverse events associated with molecular targeted therapies vary depending on the drug, but some common side effects have been observed (Launay-Vacher et al., 2015[110]). Skin toxicity is a notable adverse event associated with molecular targeted therapies. These therapies can affect normal cells and tissues in the body, including the skin, because they emphasize molecular pathways that contribute to cancer cell growth and survival (Platt and Szoka, 2008[143]). Rashes, dryness, itching, and blistering are all symptoms of skin toxicity. Managing these symptoms may necessitate the use of topical creams or dose adjustments. Another potential side effect of molecular targeted therapies is gastrointestinal toxicity. Some targeted drugs may interfere with the normal functioning of the gastrointestinal tract, causing nausea, vomiting, diarrhea, or loss of appetite. These symptoms can be distressing for patients and may necessitate supportive care measures such as antiemetic medications or dietary changes to alleviate discomfort and ensure adequate nutrition (Fischer-Cartlidge, 2014[62]).

Cardiovascular toxicity is another issue that has been raised in relation to certain molecular-targeted therapies. Some drugs used to prevent cSCC can cause problems with the cardiovascular system, such as high blood pressure, arrhythmias, or heart failure (Harwood et al., 2005[77]). It is critical that healthcare providers closely monitor patients receiving these therapies, especially those with preexisting cardiovascular conditions, and address any cardiac-related symptoms or abnormalities as soon as possible.

In addition to these specific side effects, molecular-targeted therapies can have more general side effects that are common with many cancer treatments. These symptoms could include fatigue, changes in blood cell counts, hair loss, or an increased risk of infection. Patient education and close monitoring are required to appropriately identify and manage these side effects, ensuring optimal patient outcomes and adherence to therapy (Anand et al., 2022[12]). While adverse events associated with molecular targeted therapies can be challenging, they must be balanced against the potential benefits of these therapies in preventing cSCC. When making treatment decisions, healthcare providers should carefully consider individual risk factors, treatment goals, and the overall health status of each patient. Open communication between healthcare providers and patients is critical for addressing any adverse events as soon as possible, minimizing their impact, and optimizing the overall treatment experience.

### Strategies for monitoring and managing treatment-related toxicities

The monitoring and management of treatment-related toxicities in patients receiving molecular targeted therapies to prevent cSCC calls for a proactive and multidisciplinary approach involving healthcare providers, patients, and caregivers. By implementing effective strategies, adverse events can be identified early and appropriately managed, and the overall treatment experience of patients can be enhanced. It is essential that patients and their caregivers receive comprehensive education on the potential adverse effects that may arise from molecular-targeted therapy. Patients must be informed about the common adverse effects, their expected onset and duration, and when to seek medical attention. This knowledge empowers patients to participate in their care actively, identify early warning signs, and promptly report any concerning symptoms to their healthcare provider (Liou et al., 2007[118]).

Monitoring of treatment-related toxicities depends heavily on baseline assessments. Before initiating treatment, medical professionals should conduct comprehensive evaluations of pertinent parameters, such as skin condition, cardiovascular health, gastrointestinal function, and blood cell counts (Biggins et al., 2021[25]). These baseline assessments establish a point of reference for monitoring changes and allow healthcare professionals to identify potential toxicity at an early stage The continuous monitoring is required throughout the treatment process. Close and consistent patient monitoring enables the prompt identification of adverse events. This monitoring may include periodic physical examinations, laboratory tests, and imaging studies as appropriate for the specific therapy and potential side effects. By closely monitoring patients, healthcare professionals can detect emerging toxicities and take the necessary steps to manage them effectively (Steinman et al., 2011[166]).

Establishing effective communication channels between healthcare providers, patients, and their caregivers is essential for monitoring and managing treatment-related toxicities. Patients should feel comfortable discussing their symptoms, concerns, and questions with their physician. Similarly, healthcare professionals should proactively inquire about potential adverse effects and promptly address patient concerns. Open communication ensures that adverse events are promptly reported, assessed, and managed, resulting in better patient outcomes (Steinman et al., 2011[166]). It is important to have a proactive management strategy in place when adverse events are identified. Medical professionals should choose the appropriate interventions based on the type of toxicity. These interventions may include dose adjustment, administration of supportive care measures (i.e., topical creams for skin toxicity or antiemetic medications for gastrointestinal toxicity), or referral to specialists for further evaluation and management. Regular follow-up visits are necessary for ongoing evaluation of toxicity, monitoring of treatment efficacy, and implementation of any necessary adjustments to optimize patient care.

### Recent developments in molecular targeted therapy for cSCC

The development of molecular-targeted drugs in oncology is accelerating. Due to the significance of the EGFR signaling pathway in the progression of cSCC, EGFR and its downstream effectors are the primary targets of the new therapeutic drugs currently being developed. Cetuximab is currently approved for the treatment of cSCC in patients with locally advanced tumors when combined with radiation and in patients with recurrent or metastatic cancer when combined with platinum-based chemotherapy (Bozec et al., 2013[30]). The incidence of cSCC is increasing, but treatment options for locally advanced or metastatic cancer are limited. New genomic technologies reveal high levels of mutation in the Notch gene family as well as previously identified gene variants like TP53. The mutational burden in cSCC is extremely high, making it difficult to identify driver genes and impeding the translation of genomics into clinical practice. Clinical experience with targeted therapies, such as EGFR inhibitors or immune modulatory drugs, suggests that these medications may be beneficial to patients; however, additional research into the mechanisms underlying squamous cell carcinogenesis is necessary. Radiation-induced signaling and tumor-promoting local occurrence of radiation hypersensitivity were thoroughly investigated, leading to the identification of new molecular targets. A wide range of pharmacological, biological, and immunological approaches, encompassing small molecule and antibody-based targeting methods, have been developed for use in conjunction with radiation (RT) or chemoradiation treatment (CRT). Despite a slew of encouraging experimental and preclinical results, only a small number of clinical trials have shown an improved result and/or beneficial effect for patients when RT or CRT is paired with targeted medicine (Viktorsson et al., 2023[179]). cSCC provide a challenge to doctors, and they remain difficult to treat even with a multidisciplinary approach. Because advanced tumors frequently fail to respond to traditional treatment choices, new techniques are required; yet prevention and diagnosis of early-stage tumors, which can lead to a great prognosis, are equally crucial. Most novel medicines are still in clinical trials or must be licensed before they can be used, but their therapeutic effectiveness is assured. Thus, knowing the molecular, genetic, and epigenetic factors underlying tumor cell behavior is critical for developing novel targeted medicines with minimum side effects for patients (Cozma et al., 2023[45]).

### Research and clinical translation's forthcoming directions

Future research and clinical translation of molecular targeted therapies for cSCC holds great potential for advancing treatment options and patient care. Several key directions for research and development in this field can be identified. Foremost, there is a necessity for ongoing research into the molecular changes and signaling pathways underlying cSCC. By elucidating the genetic and molecular mechanisms underlying the development and progression of cSCC, scientists can identify new potential therapeutic targets. This includes investigating novel pathways contributing to tumor growth and survival beyond commonly targeted pathways, such as EGFR and MAPK. The identification of these molecular targets can lead to the creation of new targeted therapies or the repurposing of existing agents for the treatment of cSCC. In addition, research should concentrate on overcoming resistance of cSCC to targeted therapies. Although initial responses to targeted agents can be encouraging, the emergence of resistance remains a significant problem in many instances. Understanding resistance mechanisms and exploring methods to overcome or prevent them are essential for improving long-term outcomes. To prevent or delay the emergence of resistance, this may involve combination therapies, the identification of biomarkers for predicting response and resistance, or the development of therapeutic strategies that target multiple pathways simultaneously.

Moreover, further research is necessary to refine patient selection and improve personalized treatment approaches. Clinicians can identify reliable predictive biomarkers to determine which patients are more likely to respond to specific targeted therapies. This can aid in optimizing treatment decisions by avoiding unnecessary therapies and their related toxicity. Additionally, investigating liquid biopsies or other non-invasive techniques for monitoring treatment response and detecting minimal residual disease could enhance treatment surveillance and guide clinical decision-making. It is crucial to explore the integration of targeted therapies with other treatment methods like surgery, radiation therapy, or immunotherapy. This approach can enhance the effectiveness of the treatment, overcome resistance, and improve long-term outcomes. In particular, it is essential to conduct clinical trials to investigate the synergistic effects of targeted therapies with immune checkpoint inhibitors or other immunotherapeutic agents in cSCC. Thus, it is crucial to ensure that the results of preclinical and clinical research are implemented into standard clinical practice. This can be achieved by conducting large-scale clinical trials to determine the safety and effectiveness of targeted therapies for specific patient populations. Moreover, optimizing treatment delivery and patient management strategies, such as dose optimization, treatment sequencing, and supportive care, can significantly improve clinical outcomes and enhance the patient experience.

## Conclusion

Molecular targeted therapies have the potential to significantly improve patient outcomes and enhance their quality of life in the case of cutaneous squamous cell carcinoma (cSCC). These therapies target specific molecular abnormalities, which result in improved response rates, disease control, and survival outcomes. Compared to conventional treatments, the lower toxicity and reduced side effects profile of these therapies improve the overall well-being of the patients. Moreover, the introduction of personalized medicine in this context enables treatment approaches tailored to the individual patient, optimizing therapeutic efficacy and minimizing unnecessary interventions. As research and development in this field continue, the future of molecular targeted therapies in cSCC management appears promising.

## Notes

Harpreet Singh and Mohammad Amjad Kamal (Institutes for Systems Genetics, Frontiers Science Center for Disease-Related Molecular Network, West China Hospital, Sichuan University, China; E-mail: rrs.usa.au@gmail.com) contributed equally as corresponding author.

## Declaration

### Acknowledgments

All the authors would like to acknowledge their respective institutes/organizations for providing article drafting and submission-related aids/tools.

### Author contributions

Conceptualization: HS, IS, SM; Formal analysis: SM, AK; Writing-original draft preparation: HS, MS, HC; Writing-review and editing: HS, SM, AM, MGA; Visualization: SG, AS, AM, AKM; Overall supervision, final editing, submission and correspondence: MAK. All authors have read and agreed to the published version of the manuscript.

### Funding

Not applicable.

### Institutional review board statement

Not applicable.

### Informed consent statement

Not applicable.

### Data availability statement

All relevant data has already been cited in the manuscript. No new data generated since this is a review article.

### Conflict of interest

The authors declare no conflict of interest.
